# Effect of *Crocus sativus* L. Stigmas Microwave Dehydration on Picrocrocin, Safranal and Crocetin Esters

**DOI:** 10.3390/foods10020404

**Published:** 2021-02-12

**Authors:** Aarón García-Blázquez, Natalia Moratalla-López, Cándida Lorenzo, M. Rosario Salinas, Gonzalo L. Alonso

**Affiliations:** Cátedra de Química Agrícola, E.T.S.I. Agrónomos y Montes, Universidad de Castilla-La Mancha, Campus Universitario, 02071 Albacete, Spain; Aaron.Garcia@uclm.es (A.G.-B.); Natalia.Moratalla@uclm.es (N.M.-L.); Candida.Lorenzo@uclm.es (C.L.); Rosario.Salinas@uclm.es (M.R.S.)

**Keywords:** saffron quality, secondary metabolites, drying, high performance liquid chromatography-diode array detection (HPLC-DAD), spectrophotometry

## Abstract

The dehydration process is the basis to obtain high quality saffron and to preserve it for a long time. This process modifies saffron’s main metabolites that define its quality, and are responsible for the characteristic color, taste, and aroma of the spice. In this work, the effect of microwave dehydration on saffron main metabolites (picrocrocin, safranal and crocetin esters) from *Crocus sativus* L. stigmas at three determinate powers and different time lapses was evaluated. The results showed that this dehydration process obtained similar or lower crocetin esters content, and after three months of storage, higher concentration was shown in treatments at 440 W for 36 s, 55 s, and 73 s; at 616 W for 90 s; and at 800 W for 20 s. Picrocrocin content was lower and safranal content was higher in all treatments compared to the control both before and after storage. Regarding to commercial quality, microwave dehydration obtained Category I of saffron according to International Standard Organization (ISO) 3632. After three months of storage, treatments at 616 W for 83 s and 800 W for 60 s obtained lower categories. The results obtained suggest that microwave dehydration is a suitable process for obtaining high quality saffron, 800 W with 6 lapses of 20 s being the best conditions studied.

## 1. Introduction

Saffron is the dried stigmas of *Crocus sativus* L. and its high value is due to its colour, flavour, and aroma [1]. There is confusion about the names of the spice and the plant. *C. sativus* L. is the plant, while saffron refers to the spice obtained from the dehydrated stigmas of the plant itself [2,3]. Therefore, the dehydration process is necessary to convert *C. sativus* L. stigmas into saffron.

Traditionally, saffron in Spain is obtained by *C. sativus* L. stigmas dried by a process called “toasting”, in which stigmas are put on a sieve with a silk bottom placed over a heating source, whereas in other countries, in terms of temperature, stigmas are dehydrated at room temperature under sunlight or in the shade. Stigmas’ dehydration is the key process to obtaining the spice and is responsible for saffron composition [4,5,6]. During this process, stigmas lose around 80% of their weight and, according to the International Standard ISO 3632 (2011) [7], moisture must be lower than 12% in order to preserve the spice for a long time [8]. Its quality is based on the capacity of the spice to give colour, taste, and aroma to foods and beverages, and these organoleptic properties are influenced by the dehydration process [8,9,10,11,12]. Saffron colour comes from crocetin esters also known as crocins, its bitter taste from picrocrocin, and its distinctive aroma from safranal [13,14]. Depending on the physical and chemical characteristics of saffron, it is classified into one of the three categories established by the ISO 3632 (2011) standard. This international standard is principally used to determine the saffron quality in international commercial agreements, colouring strength being the main parameter from which its price is set. Although the quality of this spice is measured via Ultraviolet-visible (UV-vis) spectrometry, according to ISO 3632 (2011), this method does not provide a precise determination of picrocrocin and safranal concentration [15].

Crocins are a group of water-soluble carotenoids responsible for saffron’s colour strength [16,17,18,19]. For each crocetin ester, there could be various geometric isomers, *trans* isomers being the most abundant and more stable than *cis* [20]. During the dehydration process, safranal is formed from picrocrocin due to high temperatures. It can also be obtained by extreme pH or a two-step enzymatic process [8,21]. Storage is also a determinant in saffron quality, as safranal concentration is higher in saffron stored longer than one month due to its formation from crocetin esters and picrocrocin during storage time [22,23].

Alternatives to traditional dehydration methods are being researched, as this process is known for being unproductive and slow [24,25]. Some techniques that are studied today are microwave dehydration, oven dehydration, freeze dehydration (lyophilization), vacuum dehydration, and infrared dehydration, among others to be applied to saffron dehydration [26]. These alternative dehydration methods show greater crocin content in freeze dried saffron and in processes where high temperature is applied [5,14]. Microwave dehydration provides great efficiency, controllability over the process, and a higher rate at a lower temperature due to water molecules absorbing the energy and evaporating quickly [13,27,28]. Compared to heat dehydration, microwave provides higher speed and volumetric heating, instead of superficial heating on account of traditional dehydration [29,30].

Previous studies have proposed microwave dehydration as a good alternative to traditional dehydration methods, as it provides higher concentrations of safranal and crocins in a shorter time under determinate conditions [5,6,14]. However, the effect of microwave dehydration on the main metabolites of saffron has not been studied in depth. Thus, the aim of this work was to study the effect of microwave dehydration on saffron’s main metabolites and its commercial quality. To achieve this, the content of the most important compounds in saffron—picrocrocin, safranal, and crocetin esters—were measured after carrying out the microwave dehydration of fresh stigmas at different powers and times, using high performance liquid chromatography-diode array detection (HPLC-DAD) and UV-vis spectrometry.

## 2. Materials and Methods

### 2.1. Samples and Reagents

Fresh stigmas of *Crocus sativus* L. were purchased from the company “Molineta de Minaya” (Minaya, Spain). The control sample consists of a part of fresh stigmas that were dehydrated according to the own company’s internal procedures, considering therefore that this saffron belongs to Protected Designation of Origin (PDO) “Azafrán de La Mancha” [4,31]. Acetonitrile used in HPLC gradient was purchased from Scharlau (Barcelona, Spain). Ultrahigh-purity water was produced using a Milli-Q system (Millipore, Bedford, MA, USA).

### 2.2. Dehydration Process

Fresh stigmas were distributed into 15 portions of 1.5 g each and placed into a filter paper box. The dehydration process was carried out at low, medium, and high power (440 W, 616 W, and 800 W, respectively) and different time lapses for each (36 s, 55 s, 73 s, and 130 s for 440 W; 26 s, 39 s, 52 s, 79 s, 83 s, and 90 s for 616 W; 20 s, 30 s, 40 s, and 60 s for 800 W) in order to control the decrease of humidity and prevent vegetal material from burning out. Between each lapse, a 10 s rest period was maintained, during which time the mass was measured to monitor weight loss. Time lapses were repeated until weight was reduced by 80% ± 2, resulting in the total energy applied at every treatment (shown in Table 1).

### 2.3. Saffron Extract Preparation

The saffron extracts were prepared according to ISO 3632 (2011) [7] slightly modified. They were then ground to a powder and passed through a sieve 0.5 mm in pore diameter; 50 mg was then placed in a 100 mL flask, adding 90 mL of Milli-Q water. The solution was stirred for 1 h at 1000 rpm using a magnetic stir bar in the dark. The flask was filled to 100 mL and homogenised through agitation. The solution was filtered through a 0.45 µm pore sized hydrophilic polytetrafluoroethylene (PTFE) filter (Millipore, Bedford, MA, USA). Two extracts were obtained from each dehydration treatment, including the control.

### 2.4. Nomenclature for Crocetin Esters

Abbreviations for crocetin esters were adopted from Carmona et al. [20]: *trans*-5-tG, *trans*-crocetin (tri-*β*-D-glucosyl)-(*β*-D-gentibiosyl) ester; *trans*-5-nG, *trans*-crocetin (*β*-D-neapolitanosyl)-(*β*-D-gentibiosyl) ester; *trans*-4-GG, *trans*-crocetin di-(*β*-D-gentibiosyl) ester; *trans*-4-ng, *trans*-crocetin (*β*-D-neapolitanosyl)-(*β*-D-glucosyl) ester; *trans*-3-Gg, *trans*-crocetin (*β*-D-glucosyl)-(*β*-D-gentibiosyl) ester; *trans*-2-gg, *trans*-crocetin di-(*β*-D-glucosyl) ester; *cis*-4-GG, *cis*-crocetin di-(*β*-D-gentibiosyl) ester; *trans*-2-G, *trans*-crocetin (*β*-D-gentibiosyl) ester; *cis*-3-Gg, *cis*-crocetin (*β*-D-glucosyl)-(*β*-D-gentibiosyl) ester; *trans*-1-g, *trans*-crocetin (*β*-D-glucosyl) ester.

### 2.5. HPLC-DAD Analysis

This analysis was performed according to the method by García-Rodríguez et al. [11]. 20 µL of each sample was injected into the Agilent 1200 HPLC chromatograph (Palo Alto, CA, USA) equipped with a 250 mm × 4.6 mm diameter, 5 µm Develosil Octadecyl System-Trifunctional (ODS-HG-5) chromatographic column (Teknokroma, Sant Cugat del Vallès, Barcelona, Spain) equilibrated at 40 °C. The eluents were water (A) and acetonitrile (B) with the following gradients: 20% B, 0–5 min; 20–80% B, 5–15 min; and 80% B, 15–20 min at 0.8 mL/min of flow rate. The DAD detector (Hewlett Packard, Waldbronn, Germany) was set at 250, 330, and 440 nm to detect picrocrocin, safranal, and crocetin esters, respectively. All analyses were performed in duplicate for each replicate (*n* = 4).

Identification of crocetin esters, picrocrocin, and safranal was carried out as previously reported [11,16]. Quantification was based on the following calibration curves [11]: C_i_ = (0.00746 ± 0.00004)A_i_ − (0.00571 ± 0.12863), correlation coefficient (R^2^) = 0.9999 for *trans*-5-tG, *trans*-5-nG, *trans*-4-GG and *trans*-4-ng; C_i_ = (0.00713 ± 0.00003)A_i_ − (0.00472 ± 0.05608), R^2^ = 0.9999 for *trans*-3-Gg, *trans*-2-gg, *trans*-2-G and *trans*-1-g; C_i_ = (0.00531 ± 0.0004)A_i_ − (0.00571 ± 0.12863), R^2^ = 0.9999 for *cis*-4-GG; C_i_ = (0.00500 ± 0.00003)A_i_ − (0.00331 ± 0.05608), R^2^ = 0.9999 for *cis*-3-Gg; C_i_ = (0.02900 ± 0.00002)A_i_ + (0.51940 ± 0.02631), R^2^ = 0.9999 for picrocrocin, and C_i_ = (0.03227 ± 0.00063)A_i_ + (0.05101 ± 0.03103), R^2^ = 0.9989 for safranal. Limits of detection (LOD) and quantification (LOQ) were taken into consideration [11].

### 2.6. UV-Vis Spectrometry

In order to perform UV-vis spectrometry analysis according to ISO 3632 (2011) [7], the same extract used for HPLC-DAD analysis was diluted to 1:10 (*v*/*v*) and then scanned by duplicate at a wavelength of 440, 330, and 257 nm by a Perkin Elmer Lambda 20 UV-Vis spectrophotometer (Perkin Elmer, Norwalk, CT, USA) in a 1 cm pathway quartz cell.

### 2.7. Statistical Analysis

The statistical analysis was performed using SPSS Statistics 25 for Windows (IBM, Armond, NY, USA). Data were analysed by performing one-way analysis of variance (ANOVA), with Duncan’s test for multiple comparisons and Dependent *t*-test for paired samples with degree of freedom of 3, considering *p* < 0.05 as statistically significant (95% confidence interval).

## 3. Results and Discussion

### 3.1. Content of the Main Metabolites of Microwave-Dehydrated Saffron

To determine the effect of microwave dehydration on saffron’s main metabolites, the stigmas were dehydrated at different powers and time lapses, and subsequently analysed through HPLC-DAD, which is the only way to determine these compounds [11].

Picrocrocin, safranal, and crocetin esters concentrations are shown in Table 2. With regards to picrocrocin, the first metabolite to be detected in the analysis, control values were significantly higher in traditional dehydration than the microwave treatments. Safranal was not quantified in traditional “toasting” because its content was below the limit of quantification. However, all microwave dehydration treatments showed safranal. Tong et al. [6] observed that an increase in the time of microwave dehydration of fresh stigmas (from 3 to 6 min) at 600 W registered a decrease of safranal concentration, while at 450 W a longer time (from 6 to 10 min) obtained a higher concentration. In our study, it is essential to supply an energy of the same order by combining powers and times (providing different time lapses), so that within each power studied there is no great difference in total time to be able to compare our results with those showed by these authors. Another study described the effect on different dehydration methods, including microwave dehydration [14], in which safranal reported the highest concentration in compared to electric oven dehydration and vacuum oven dehydration; however, the results in the mentioned study cannot be compared to ours as the dehydration time employed was excessive (1.9 h).

The total content of crocetin esters showed significant variances between the control and the other microwave treatments, except for 440-36, 440-130, 616-39, 616-52, and 800-20. This also happened in the sum of *trans* crocetin esters with the exception for the treatment 440-130, which also showed significant differences to the control. The *cis* isomers showed significant differences in microwave-treated samples against the control, and those samples which showed the highest concentration of *cis* isomers were also the ones that had the highest safranal concentration. In this sense, Carmona et al. [23] reported that a high temperature for the dehydration process promotes the isomerization of *trans* crocetin esters to *cis*, as well as safranal synthesis from these carotenoids and picrocrocin. Speranza and Dadá [32] observed the formation of 13-*cis*-crocin, after 1 h of exposition to light. On the other hand, this is the first time that the concentration of the main metabolites of stigmas dehydrated by microwave is evaluated in detail. There is no previous study showing the concentration of *trans* and *cis* isomers, which could be used to compare our results, but it seems that the energy supplied by microwave dehydration of stigmas may be involved in the formation of *cis* crocetin esters. Thus, all these results could indicate that different energy sources could influence the isomerization process and the formation of safranal from picrocrocin and the cycling of *cis* isomers.

The proportion between *trans* and *cis* crocetin esters was analysed, showing that *trans* crocetin esters are the predominant form of crocins. All the microwave-treated samples showed significant variance with the control, the values of which ranged from 20 in 800-40 to 48 in 616-83. Therefore, the saffron obtained by microwave dehydration contains less content of *trans* crocetin esters than those obtained from traditional “toasting”, resulting in saffron with less bioactive capacity, as *trans* crocetin esters compounds are more bioactive than *cis* isomers [3,12,33].

Crocins are a wide group of glycosyl esters, of which the predominant ones are *trans*-4-GG and *trans*-3-Gg. Concentration values of the crocetin esters are shown in Table 3. One of the predominant crocetin esters is *trans*-4-GG, which showed differences to the control in all microwave dehydration treatments, except for 440-73, 616-83, 616-90, and 800-40. The other main crocetin ester is *trans*-3-Gg, which showed the highest concentration values of all the reported glycosides and obtained significant differences between the control and all the microwave dehydration treatments studied. Previous studies showed that *trans*-4-GG concentration is higher than *trans*-3-Gg in traditionally dehydrated stigmas [11,16,34,35]. Other works are in accordance with our results as a greater value of *trans*-3-Gg concentration than *trans*-4-GG is also registered when stigmas are dried in the shade or freeze-dried [35,36]. However, under these different methods of dehydration, contrary results have also been obtained [36,37]. Therefore, it can be mentioned that these two compounds are the main crocetin esters of saffron.

In the other crocetin esters studied, significant differences to the control were shown for *trans*-5-nG, *trans*-2-gg, *trans*-2-G and *trans*-1-g. All these compounds showed higher content in control except for *trans*-1-g, whose content was higher in all the microwave treatments [3].

*Cis*-4-GG and *cis*-3-Gg were the *cis*-crocetin esters identified and quantified in this work. Both showed significant differences to the control with higher content in the treatments studied. These crocetin esters are known for being less bioactive than the *trans* esters, which results in a saffron with lower bioactive capacity. 

Saffron aroma is enhanced after at least a month of its storage in stigmas traditionally dehydrated, the reason why saffron is not sold immediately after dehydration. In addition, previous studies observed that saffron’s main compounds’ content evolves over time [4,35]. Thus, microwave dehydrated saffron was stored for three months in order to study the evolution of the main metabolites, after which these compounds were analysed by HPLC-DAD again.

Picrocrocin, safranal, and total crocetin esters concentration after three months of storage are shown in Table 4. Both picrocrocin and safranal maintained the significant differences shown between the control and the treatments studied before the storage. In practically all dehydration parameters studied, safranal values were higher after storage, which matches with the previous results of storage effect on safranal according to Maggi et al. [38]. Moreover, Sereshti et al. [39] reported that safranal content is lower in freshly dried stigmas, and at least a month of storage is known to be necessary for the development of saffron aroma [22,23].

The content of total crocetin esters was less in the control and in some microwave dehydration treatments compared to the results obtained before its storage. The treatment 616-83 showed significant variances with the rest of the microwave dehydration treatments and with the control for total crocetin esters and for the sum of *trans* crocetin esters. All dehydration treatments showed significantly higher *cis* content than the control, resulting in a lower *trans*/*cis* proportion. The treatments that showed the highest concentration of total *cis* isomers also registered the highest safranal content, which was also observed before the storage, reinforcing the previously mentioned relationship between safranal and *cis* crocetin esters.

Crocetin esters were analysed after three months of storage to analyse whether deterioration of these compounds had taken place. Main crocetin esters’ concentration values are shown in Table 5.

The two main crocetin esters, *trans*-4-GG and *trans*-3-Gg kept the previous proportion, *trans*-3-Gg concentration being higher than *trans*-4-GG in all microwave dehydration treatments studied and in the control. The concentration value of *trans*-4-GG compared to the content obtained before storage decreased slightly in the control, while it increased in some of the microwave dehydration treatments. For *trans*-3-Gg, the control also obtained lower value compared to its content before storage, and the treatments increased their concentration only in some of them. Regarding *trans*-4-GG, at 440 W for 36 s, 55 s, and 73 s, along with 616-90 and 800-20, there was significant variance compared to the rest of the treatments. *Trans*-3-Gg showed similar significant differences to those described for *trans*-4-GG in relation to the microwave dehydration treatments. As before the storage of the saffron, *cis*-4-GG and *cis*-3-Gg were identified and quantified. Regarding *cis*-4-GG, all microwave treated samples showed significant variances with the control, which obtained the lowest concentration (1.04 g/kg). *Cis*-3-Gg, however, could not be detected in the control and in the treatments performed at 440 W.

Therefore, after storage, new significant variances were observed. Some of the microwave treated samples presented different trends compared to the control. It is noteworthy that microwave dehydrated saffron showed higher total crocetin esters content in some treatments after storage (at 440 W for 36 s, 55 s and 73 s, 616-90 and at 800 W for 20 s, 40 s and 60 s) and safranal content decreased in 440-73, although the storage is known for improving safranal content but also diminishing carotenoids due to its oxidation [23,24,40]. Considering the main metabolites’ content of saffron obtained from the different microwave dehydration treatments studied, and compared to the control (traditionally obtained saffron), the treatments that obtained an increase in total crocetin esters after storage also showed high contents of picrocrocin and safranal. These treatments were able to dehydrate stigmas of *C. sativus* L. and obtain saffron with a content of main metabolites equal to or superior to those obtained by traditionally dehydrated saffron. Among them, treatment 800-20 stands out for obtaining saffron with the highest content of picrocrocin, safranal, and total crocetin esters. The 440-130 treatment would stand out for obtaining saffron with a great bioactive capacity. In addition, it would have high content of the main metabolites. On the other hand, 616-83 would not be recommended for use due to the lowest content of all evaluated compounds in the saffron obtained from this treatment.

**Figure 1:**
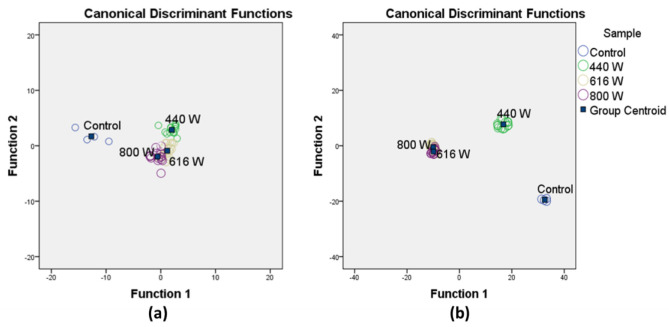
Graphical plot of the results from the stepwise canonical analysis of saffron samples obtained by microwave dehydration at different powers and control samples, before (**a**) and after three months of storage (**b**).

Discriminant function analysis was performed on results from HPLC-DAD grouped together according to the power used in the dehydration process, in order to identify a relationship between the compounds during the dehydration process and determine canonical functions that separate samples within two functions (Figure 1).

After the dehydration process, the control sample was separated from microwave dehydrated stigmas by function 1 (71.4% of variance) and function 2 (92.8% of cumulative variance) (Figure 1a). Function 1 depended on *trans*-4-GG, *trans*-3-Gg, and *trans*-5-nG mainly, and function 2 depended on *trans*-4-GG, *trans*-5-tG, and *trans*-2-G primarily. Similar analysis was performed after three months of storage, and in this case, control sample and stigmas dehydrated at 440 W were separated from the rest of the treatments by function 1 (82.9% of variance) and function 2 (99.5% of cumulative variance) (Figure 1b). In this case, function 1 depended on *cis*-3-Gg, *trans*-4-GG, and *trans*-2-G mainly, and function 2 depended on *trans*-3-Gg, *trans*-5-tG, and *trans*-5-nG, principally. After three months of storage, a higher separation of the treatments at 440 W due to *cis*-3-Gg, mainly, was observed. Considering Table 3 and Table 5, *cis*-3-Gg content decreased at 440 W after storage, while the isomer *trans*-3-Gg increased. It seems that the dehydration process at this power produces a less stable isomerization than the rest of the powers studied.

Therefore, the microwave dehydration process separated saffron obtained from the stigmas dehydrated by “toasting”, and this separation was more pronounced after three months of storage, due mainly to the content of crocetin esters and more specifically to the most abundant crocins.

### 3.2. Commercial Quality of Microwave-Dehydrated Saffron

The commercial quality of saffron is classified into three categories established by ISO 3632 [7]. This standard indicates that the highest quality category must be composed of saffron with a minimum value of 200 of $A_{1\;cm}^{1\%}$ 440 nm, 70 of $A_{1\;cm}^{1\%}$ 257 nm, and a $A_{1\;cm}^{1\%}$ 330 nm value between 20 and 50. Results of UV-vis spectrophotometric analyses of the control and saffron dehydrated at different powers and time lapses are shown in Table 6.

The initial results showed that the control could not be classified as saffron according to ISO 3632 [7] due to its low value of $A_{1\;cm}^{1\%}$ 330 nm, while all the microwave treatments produced saffron belonging to Category I. Color strength ($A_{1\;cm}^{1\%}$ 440 nm) results showed that there were several differences across different treatments, although saffron obtained in 440-73, 616-83, 616-90, 800-30, 800-40, and 800-60 did not show significant differences between traditional and microwave dehydration. Values of ISO parameters obtained in microwave dehydration treatments are in concordance with those reported by Maghsoodi [5] in a previous essay, taking into account their results obtained at 200 W for 720 s (in total), since the same order of energy (144,000 J) as our work was applied. The values of $A_{1\;cm}^{1\%}$ 257 nm were significantly lower for all the microwave dehydration treatments compared to the control.

After storage, the treatments 616-83 and 800-60 showed a $A_{1\;cm}^{1\%}$ 440 nm value below 200, which relegated them to Category III (≤170) and II (≤200), respectively. These treatments did not maintain the quality previously observed before storage, which shows that they are not suitable for producing high quality saffron.

Saffron value is mainly determined by its $A_{1\;cm}^{1\%}$ 440 nm value, therefore, producers want the saffron with the highest 440 nm value as possible. Between all the treatments analyzed, those that showed the highest $A_{1\;cm}^{1\%}$ 440 nm value were 440-55, 440-73, 616-90, and 800-20. The last mentioned showed high crocetin esters content in HPLC-DAD analysis, proving to be a good alternative to traditional “toasting” for obtaining high quality saffron.

## 4. Conclusions

We demonstrated that microwave dehydration produces saffron with similar content of crocetin esters and more safranal than saffron obtained from “toasting” under conditions used in 800-20. This treatment maintained high metabolite content after three months of storage; therefore, microwave dehydration provides saffron with high metabolite content and better preservation.

Saffron’s commercial quality was measured by UV-vis spectrophotometric analysis in order to classify the saffron obtained into ISO 3632 categories. Between all the treatments that produced saffron belonging to ISO Category I, 800-20 was the one that obtained the highest $A_{1\;cm}^{1\%}$ 440 nm value. The results indicate that saffron obtained from microwave dehydration is an adequate alternative to “toasting” because of the high metabolite content produced and the higher commercial quality obtained.

## Figures and Tables

**Table 1:** Dehydration treatments applied to fresh stigmas of *Crocus sativus* L.

Treatment	Power (W)	Seconds (s)/Lapse	N° Lapses	Total Time (s)	Joules (J)
440-36	440	36	6	216	95,040
440-55	440	55	4	220	96,800
440-73	440	73	3	219	96,360
440-130	440	130	2	260	114,400
616-26	616	26	6	156	96,096
616-39	616	36	4	144	88,704
616-52	616	52	3	156	96,096
616-79	616	78	2	156	96,096
616-83	616	83	2	166	102,256
616-90	616	90	2	180	110,880
800-20	800	20	6	120	96,000
800-30	800	30	5	150	120,000
800-40	800	40	3	120	96,000
800-60	800	60	2	120	96,000

**Table 2:** Content of the main compounds of saffron obtained under different microwave dehydration treatments.

Treatment	Compounds (g/kg Saffron ± SD)					
	Picrocrocin	Safranal	Total CE	∑ *Trans*-CE	∑ *Cis*-CE	*Trans*/*Cis*
Control	244.2 ± 8.9 i	<LOQ	262.7 ± 8.7 f	260.4 ± 8.9 f	2.35 ± 0.17 a	113 ± 12 g
440-36	215.4 ± 0.1 e–g	0.41 ± 0.03 ab	252.0 ± 1.7 ef	245.3 ± 1.6 d–f	6.70 ± 0.14 de	37 ± 1 de
440-55	192.6 ± 3.2 bc	0.71 ± 0.07 bc	239.1 ± 6.4 b–e	231.8 ± 6.1 b–e	7.30 ± 0.37 e	32 ± 1 cd
440-73	174.6 ± 2.9 a	1.97 ± 0.15 f	226.6 ± 2.8 a–c	216.3 ± 2.6 ab	10.35 ± 0.36 fg	21 ± 1 ab
440-130	201.3 ± 0.4 cd	0.40 ± 0.01 ab	248.9 ± 1.5 d–f	242.4 ± 1.5 c–e	6.43 ± 0.05 de	38 ± 1 d–f
616-26	208.4 ± 3.8 d–f	0.90 ± 0.11 cd	243.4 ± 7.6 c–e	235.6 ± 7.6 c–e	7.72 ± 0.10 e	31 ± 1 b–d
616-39	217.9 ± 0.4 fg	1.39 ± 0.20 e	250.9 ± 3.9 d–f	244.2 ± 3.9 d–f	6.71 ± 0.28 de	37 ± 2 de
616-52	221.7 ± 2.2 g	1.34 ± 0.15 e	255.2 ± 4.0 ef	248.7 ± 4.2 ef	6.53 ± 0.26 de	38 ± 2 d–f
616-79	202.6 ± 2.0 cd	0.23 ± 0.02 a	231.0 ± 6.0 a–c	225.1 ± 5.8 a–c	5.94 ± 0.17 cd	38 ± 1 d–f
616-83	177.8 ± 2.7 a	0.21 ± 0.03 a	216.9 ± 6.0 a	212.5 ± 5.9 a	4.47 ± 0.30 b	48 ± 3 f
616-90	174.3 ± 2.0 a	0.41 ± 0.04 ab	221.6 ± 3.8 ab	214.7 ± 3.6 ab	6.93 ± 0.19 de	31 ± 2 b–d
800-20	233.7 ± 2.7 h	1.15 ± 0.14 de	252.7 ± 8.5 ef	245.7 ± 8.2 d–f	7.00 ± 0.37 de	35 ± 1 e
800-30	207.3 ± 0.7 de	2.25 ± 0.22 f	238.3 ± 3.6 b–e	228.4 ± 3.6 a–d	9.97 ± 0.78 f	23 ± 2 a–c
800-40	202.2 ± 0.2 cd	2.69 ± 0.35 g	227.5 ± 4.1 a–c	216.2 ± 4.5 ab	11.36 ± 0.99 g	20 ± 2 a
800-60	188.7 ± 4.4 b	0.38 ± 0.05 ab	233.4 ± 6.6 a–d	228.4 ± 6.4 a–d	4.97 ± 0.19 bc	46 ± 1 ef

LOQ = limit of quantification. Total CE = Total crocetin esters. ∑ *trans*-CE = sum of *trans* isomers of crocetin esters. ∑ *cis*-CE = sum of *cis* isomers of crocetin esters. Values are the mean of two extracts conducted in duplicate (2 × 2 n), SD = standard deviation. One-way analysis of variance (ANOVA) for each column is included. Different letters within each column represent statistically significant variances, according to Duncan test (*p* < 0.05).

**Table 3:** : Crocetin esters content of saffron obtained by different dehydration treatments.

Treatment	Compounds (g/kg Saffron ± SD)									
	*Trans*-5-tG	*Trans*-5-nG	*Trans*-4-GG	*Trans*-4-ng	*Trans*-3-Gg	*Trans*-2-gg	*Cis*-4-GG	*Trans*-2-G	*Cis*-3-Gg	*Trans*-1-g
Control	0.33 ± 0.09 ab	1.18 ± 0.12 e	90.2 ± 4.3 a	1.37 ± 0.15 a	125.8 ± 4.5 d	39.11 ± 4.62 d	1.39 ± 0.11 a	2.26 ± 0.57 b	0.96 ± 0.08 a	0.16 ± 0.01 a
440-36	0.83 ± 0.02 f	0.37 ± 0.02 a–d	107.9 ± 0.6 e	2.08 ± 0.16 bc	113.1 ± 1.9 c	20.62 ± 0.32 a-c	4.20 ± 0.06 de	<LOD	2.50 ± 0.09 d-f	0.42 ± 0.01 g
440-55	0.45 ± 0.05 a–c	0.24 ± 0.03 a–c	97.5 ± 1.3 bc	1.63 ± 0.16 ab	110.8 ± 3.9 c	20.74 ± 1.36 a–c	4.50 ± 0.18 de	<LOD	2.80 ± 0.19 fg	0.46 ± 0.01 h
440-73	0.38 ± 0.04 ab	0.14 ± 0.04 a	94.6 ± 0.4 ab	1.85 ± 0.20 a–c	97.5 ± 2.0 a	21.38 ± 0.38 bc	6.49 ± 0.18 f	<LOD	3.86 ± 0.18 h	0.46 ± 0.00 h
440-130	0.73 ± 0.01 ef	0.33 ± 0.02 a–d	104.9 ± 0.1 de	2.32 ± 0.13 c	111.6 ± 1.3 c	21.48 ± 0.20 bc	3.98 ± 0.03 cd	0.53 ± 0.02 a	2.45 ± 0.01 d–f	0.54 ± 0.01 i
616-26	0.67 ± 0.07 ef	0.43 ± 0.09 cd	101.4 ± 2.2 cd	1.54 ± 0.13 ab	108.3 ± 4.1 bc	22.17 ± 1.02 c	5.13 ± 0.14 e	0.73 ± 0.17 a	2.59 ± 0.05 ef	0.37 ± 0.01 f
616-39	0.71 ± 0.06 ef	0.43 ± 0.07 cd	105.5 ± 0.6 de	1.75 ± 0.19 a–c	112.0 ± 2.8 c	22.82 ± 0.57 c	4.47 ± 0.21 de	0.72 ± 0.20 a	2.24 ± 0.12 c–e	0.29 ± 0.01 d
616-52	0.71 ± 0.04 ef	0.46 ± 0.08 cd	107.6 ± 1.6 e	2.07 ± 0.30 bc	113.9 ± 2.8 c	23.00 ± 0.47 c	4.31 ± 0.22 de	0.60 ± 0.12 a	2.23 ± 0.10 c–e	0.33 ± 0.01 e
616-79	0.67 ± 0.04 ef	0.46 ± 0.03 cd	101.5 ± 1.7 cd	1.54 ± 0.04 ab	100.5 ± 3.2 ab	19.31 ± 0.97 a–c	3.88 ± 0.10 cd	0.72 ± 0.02 a	2.06 ± 0.07 cd	0.34 ± 0.01 e
616-83	0.44 ± 0.04 a–c	0.20 ± 0.05 ab	95.7 ± 1.8 a–c	1.37 ± 0.14 a	97.4 ± 3.3 a	16.62 ± 0.90 a	2.86 ± 0.19 b	0.45 ± 0.04 a	1.61 ± 0.12 b	0.32 ± 0.00 e
616-90	0.30 ± 0.02 a	0.17 ± 0.02 a	94.3 ± 1.0 ab	1.37 ± 0.07 a	100.5 ± 2.2 ab	17.09 ± 0.49 ab	4.33 ± 0.08 de	0.44 ± 0.02 a	2.60 ± 0.11 ef	0.55 ± 0.05 i
800-20	0.64 ± 0.06 de	0.56 ± 0.10 d	105.2 ± 2.9 de	1.84 ± 0.27 a–c	114.9 ± 4.2 c	21.89 ± 1.10 c	4.66 ± 0.25 de	0.43 ± 0.06 a	2.33 ± 0.14 de	0.22 ± 0.01 b
800-30	0.50 ± 0.04 b–d	0.42 ± 0.10 b–d	98.4 ± 1.0 bc	1.75 ± 0.20 a–c	105.1 ± 2.2 a–c	21.42 ± 0.40 bc	6.93 ± 0.76 fg	0.52 ± 0.13 a	3.04 ± 0.02 g	0.25 ± 0.01 c
800-40	0.40 ± 0.08 ab	0.35 ± 0.08 a–d	95.2 ± 1.7 ab	1.70 ± 0.22 ab	96.4 ± 2.6 a	21.11 ± 0.33 a–c	7.66 ± 0.70 g	0.80 ± 0.27 a	3.70 ± 0.37 h	0.25 ± 0.01 c
800-60	0.60 ± 0.06 c–e	0.33 ± 0.08 a–d	100.3 ± 1.7 b–d	1.49 ± 0.14 ab	104.8 ± 3.9 a–c	20.26 ± 0.72 a–c	3.14 ± 0.14 bc	0.39 ± 0.02 a	1.83 ± 0.05 bc	0.26 ± 0.00 c

LOD: limit of detection. *trans*-5-tG: *trans*-crocetin (tri-*β*-D-glucosyl)-(*β*-D-gentibiosyl) ester; *trans*-5-nG: *trans*-crocetin (*β*-D-neapolitanosyl)-(*β*-D-gentibiosyl) ester; *trans*-4-GG: *trans*-crocetin di-(*β*-D-gentibiosyl) ester; *trans*-4-ng: *trans*-crocetin (*β*-D-neapolitanosyl)-(*β*-D-glucosyl) ester; *trans*-3-Gg: *trans*-crocetin (*β*-D-glucosyl)-(*β*-D-gentibiosyl) ester; *trans*-2-gg: *trans*-crocetin di-(*β*-D-glucosyl) ester; *cis*-4-GG: *cis*-crocetin di-(*β*-D-gentibiosyl) ester; *trans*-2-G: *trans*-crocetin (*β*-D-gentibiosyl) ester; *cis*-3-Gg: *cis*-crocetin (*β*-D-glucosyl)-(*β*-D-gentibiosyl) ester; *trans*-1-g: *trans*-crocetin (*β*-D-glucosyl) ester; Values are the mean of two extracts conducted in duplicate (2 × 2 n), SD = standard deviation. One-way analysis of variance (ANOVA) for each column is included. Different letters within each column represent statistically significant variances, according to Duncan test (*p* < 0.05).

**Table 4:** Content of the main compounds of saffron obtained under different dehydration treatments after being stored for three months.

Treatment	Compounds (g/kg Saffron ± SD)					
	Picrocrocin	Safranal	Total CE	∑ *Trans*-CE	∑ *Cis*-CE	*Trans*/*Cis*
Control	247.3 ± 3.9 g *	0.33 ± 0.11 a	232.2 ± 8.5 de	231.2 ± 8.5 ef	1.04 ± 0.03 a	223 ± 1 h
440-36	212.5 ± 2.1 e	0.88 ± 0.01 bc	268.2 ± 1.5 g	264.3 ± 1.6 hi	3.90 ± 0.14 c	68 ± 3 f
440-55	209.4 ± 1.0 e	0.81 ± 0.00 b	266.6 ± 7.2 g	262.8 ± 6.9 hi	3.76 ± 0.29 c	71 ± 4 f
440-73	195.0 ± 0.2 e	1.59 ± 0.00 f	254.9 ± 0.4 g	249.9 ± 0.3 i	5.03 ± 0.02 e	50 ± 1 e
440-130	186.6 ± 2.5 b	1.24 ± 0.02 d	238.8 ± 2.7 e	235.9 ± 2.5 fg	2.90 ± 0.16 b	82 ± 4 g
616-26	208.4 ± 0.1 de *	0.98 ± 0.01 c *	226.3 ± 1.0 c–e	219.0 ± 0.8 c–e	7.28 ± 0.19 f	30 ± 1 bc *
616-39	203.1 ± 1.3 c–e	1.76 ± 0.02 g	215.2 ± 2.6 c	207.3 ± 2.4 c	7.87 ± 0.12 f	26 ± 0 b
616-52	197.8 ± 0.5 b–d	1.65 ± 0.01 fg	220.6 ± 0.5 cd	212.8 ± 0.4 cd	7.79 ± 0.16 f	27 ± 1 b
616-79	189.0 ± 0.7 b	0.78 ± 0.00 b	174.1 ± 4.9 b	169.7 ± 4.8 b	4.40 ± 0.04 cd	39 ± 1 d *
616-83	158.1 ± 2.5 a	1.45 ± 0.02 e	130.8 ± 1.7 a	126.7 ± 1.5 a	4.07 ± 022 cd *	31 ± 1 bc
616-90	197.4 ± 0.1 bc	1.18 ± 0.00 d	275.1 ± 0.5 fg	267.1 ± 0.5 gh	7.77 ± 0.12 f	34 ± 1 bc *
800-20	235.6 ± 7.0 f *	1.59 ± 0.29 f	262.0 ± 3.3 g *	252.5 ± 3.3 hi *	9.54 ± 0.07 g	26 ± 0 b
800-30	205.7 ± 2.9 de *	3.59 ± 0.04 h	241.6 ± 3.6 ef *	228.2 ± 3.1 d–f *	13.37 ± 0.51 h	17 ± 0 a
800-40	193.2 ± 1.7 bc	3.82 ± 0.09 i	233.8 ± 3.5 de *	218.9 ± 3.0 c–e *	14.85 ± 0.51 i	15 ± 0 a
800-60	161.5 ± 1.8 a	1.42 ± 0.04 e	164.4 ± 0.7 b	159.6 ± 0.7 b	4.81 ± 0.04 d *	33 ± 0 c

Total CE: Total crocetin esters. ∑ *trans*-CE: sum of *trans* isomers of crocetin esters. ∑ *cis*-CE: sum of *cis* isomers of crocetin esters. Values are the mean of two extracts conducted in duplicate (2 × 2 n), SD = standard deviation. One-way analysis of variance (ANOVA) for each column is included. Different letters within each column represent statistically significant variances, according to Duncan test (*p* < 0.05). Data marked with * does not show significant variances to same treatment and compound before storage (*p* < 0.05).

**Table 5:** Crocetin esters content of saffron obtained under different dehydration treatments after being stored for three months.

Treatment	Compounds (g/kg Saffron ± SD)									
	*Trans*-5-tG	*Trans*-5-nG	*Trans*-4-GG	*Trans*-4-ng	*Trans*-3-Gg	*Trans*-2-gg	*Cis*-4-GG	*Trans*-2-G	*Cis*-3-Gg	*Trans*-1-g
Control	0.43 ± 0.03 bc *	1.44 ± 0.02 i	90.0 ± 1.1 c *	1.20 ± 0.07 c *	110.4 ± 6.2 ef	4.75 ± 0.73 d	1.04 ± 0.10 a	21.32 ± 0.26 g	<LOD	1.67 ± 0.18 d
440-36	0.85 ± 0.02 f *	0.74 ± 0.04 gh	115.0 ± 1.1 g	1.80 ± 0.12 de *	121.7 ± 0.8 g	2.88 ± 0.33 c	3.90 ± 0.14 c	20.16 ± 0.11 g	<LOD	1.24 ± 0.05 bc
440-55	0.77 ± 0.02 ef	0.69 ± 0.02 f–h	114.6 ± 1.2 g	2.02 ± 0.10 de	122.9 ± 4.5 g	2.53 ± 0.20 bc	3.76 ± 0.29 c	18.25 ± 0.88 f	<LOD	1.11 ± 0.09 b
440-73	0.72 ± 0.00 f	0.57 ± 0.00 gh	110.2 ± 2.9 g	2.22 ± 0.01 f	111.5 ± 0.2 g	2.36 ± 0.01 c	5.03 ± 0.02 f	21.05 ± 0.04 h	<LOD	1.28 ± 0.00 c
440-130	0.65 ± 0.01 df	0.58 ± 0.02 ef	105.3 ± 0.4 f *	1.86 ± 0.02 de	107.4 ± 1.7 de	2.52 ± 0.10 bc	2.90 ± 0.16 b	16.53 ± 0.32 e	<LOD	1.05 ± 0.05 b
616-26	0.45 ± 0.04 bc	0.36 ± 0.02 bc *	99.3 ± 1.2 de *	2.36 ± 0.23 f	103.9 ± 1.1 c–e *	2.36 ± 0.05 a–c	4.37 ± 0.11 cd	9.10 ± 0.43 c	2.91 ± 0.09 c	1.17 ± 0.08 bc
616-39	0.37 ± 0.01 b	0.36 ± 0.02 bc *	95.2 ± 0.4 d	1.93 ± 0.27 de *	97.5 ± 1.5 c	2.14 ± 0.06 a–c	5.04 ± 0.05 e	8.70 ± 0.32 c	2.83 ± 0.06 c	1.07 ± 0.04 b
616-52	0.48 ± 0.06 bc	0.44 ± 0.01 cd*	97.0 ± 0.6 de	2.11 ± 0.06 ef *	100.3 ± 1.5 cd	2.31 ± 0.05 a–c	4.89 ± 0.17 de	9.05 ± 0.09 c	2.90 ± 0.02 c	1.11 ± 0.02 b
616-79	0.09 ± 0.04 a	0.24 ± 0.02 ab	78.1 ± 2.5 b	1.85 ± 0.01 de	82.5 ± 2.1 b	1.85 ± 0.01 ab	2.45 ± 0.02 b	4.32 ± 0.26 ab	1.95 ± 0.05 b *	0.73 ± 0.01 a
616-83	<LOQ	<LOQ	60.2 ± 0.3 a	0.47 ± 0.04 a	60.9 ± 1.0 a	1.73 ± 0.12 a	2.43 ± 0.15 b	2.81 ± 0.33 a	1.64 ± 0.07 a *	0.58 ± 0.09 a
616-90	0.82 ± 0.02 de	0.82 ± 0.02 e–g	118.3 ± 0.2 g	1.72 ± 0.01 d	128.2 ± 0.3 g	2.48 ± 0.02 a–c	4.16 ± 0.03 cd	13.48 ± 0.01 d	3.81 ± 0.00 d	1.28 ± 0.01 bc
800-20	0.83 ± 0.07 f	0.83 ± 0.07 h	112.8 ± 1.5 g	1.98 ± 0.05 de *	118.8 ± 1.8 fg *	2.73 ± 0.06 c	5.78 ± 0.04 f	13.25 ± 0.08 d	3.76 ± 0.02 d	1.30 ± 0.05 bc
800-30	0.53 ± 0.03 cd *	0.53 ± 0.03 de *	101.4 ± 0.4 ef	1.88 ± 0.14 de *	104.6 ± 1.8 c–e *	2.47 ± 0.11 a–c	9.22 ± 0.38 g	15.14 ± 0.60 e	4.14 ± 0.12 e	1.70 ± 0.08 d
800-40	0.43 ± 0.01 bc *	0.43 ± 0.01 cd *	96.5 ± 0.4 de *	1.38 ± 0.08 c	99.9 ± 1.7 cd *	2.48 ± 0.11 a–c	10.53 ± 0.38 h	16.02 ± 0.66 e	4.32 ± 0.13 e	1.82 ± 0.11 d
800-60	0.15 ± 0.05 a	0.15 ± 0.05 a	76.4 ± 0.3 b	0.90 ± 0.04 b	74.6 ± 0.3 b	1.82 ± 0.06 ab	2.76 ± 0.01 b	4.85 ± 0.17 b	2.05 ± 0.03 b	0.66 ± 0.02 a

LOQ: limit of quantification; LOD: limit of detection. *trans*-5-tG: *trans*-crocetin (tri-*β*-D-glucosyl)-(*β*-D-gentibiosyl) ester; *trans*-5-nG: *trans*-crocetin (*β*-D-neapolitanosyl)-(*β*-D-gentibiosyl) ester; *trans*-4-GG: *trans*-crocetin di-(*β*-D-gentibiosyl) ester; *trans*-4-ng: *trans*-crocetin (*β*-D-neapolitanosyl)-(*β*-D-glucosyl) ester; *trans*-3-Gg: *trans*-crocetin (*β*-D-glucosyl)-(*β*-D-gentibiosyl) ester; *trans*-2-gg: *trans*-crocetin di-(*β*-D-glucosyl) ester; *cis*-4-GG: *cis*-crocetin di-(*β*-D-gentibiosyl) ester; *trans*-2-G: *trans*-crocetin (*β*-D-gentibiosyl) ester; *cis*-3-Gg: *cis*-crocetin (*β*-D-glucosyl)-(*β*-D-gentibiosyl) ester; *trans*-1-g: *trans*-crocetin (*β*-D-glucosyl) ester; Values are the mean of two extracts conducted in duplicate (2 × 2 n), SD = standard deviation. One-way analysis of variance (ANOVA) for each column is included. Different letters within each column represent statistically significant variances, according to Duncan test (*p* < 0.05). Data marked with * did not show significant variance with same treatment and compound before storage (*p* < 0.05).

**Table 6:** UV-vis spectrophotometric parameter values of saffron samples obtained under different microwave dehydration treatments.

Treatment	($A_{1\;cm}^{1\%}$ ± SD) Initial				($A_{1\;cm}^{1\%}$ ± SD) after Storage			
	440 nm	330 nm	257 nm	Cat.	440 nm	330 nm	257 nm	Cat.
Control	238 ± 8 ab	15 ± 1 a	146 ± 24 b	-	235 ± 12 c	22 ± 1 ab	97 ± 3 e–h	I
440-36	260 ± 3 de	24 ± 1 b–d	96 ± 1 a	I	262 ± 3 ef	26 ± 1 b–e	97 ± 1 e–h	I
440-55	256 ± 4 cd	25 ± 1 b–e	96 ± 2 a	I	268 ± 3 fg	26 ± 1 b–e	100 ± 1 gh	I
440-73	239 ± 4 ab	25 ± 2 de	88 ± 2 a	I	259 ± 1 g	27 ± 1 c–e	95 ± 1 e–g	I
440-130	265 ± 1 d–f	28 ± 1 f	99 ± 1 a	I	246 ± 2 c–e	24 ± 1 bc	87 ± 1 c	I
616-26	256 ± 5 cd	25 ± 1 c–e	96 ± 2 a	I	248 ± 1 c–e	19 ± 1 a	90 ± 1 cd	I
616-39	269 ± 2 ef	25 ± 2 de	100 ± 1 a	I	262 ± 2 ef	30 ± 2 ef	99 ± 3 f–h	I
616-52	274 ± 3 f	26 ± 1 ef	102 ± 1 a	I	258 ± 2 d–f	25 ± 1 b–d	94 ± 2 d–f	I
616-79	259 ± 1 de	28 ± 1 f	95 ± 1 a	I	246 ± 1 c–e	29 ± 1 de	93 ± 2 de	I
616-83	237 ± 2 ab	23 ± 1 bc	87 ± 1 a	I	156 ± 1 a	35 ± 1 g	82 ± 1 b	III
616-90	235 ± 1 a	26 ± 1 d–f	89 ± 1 a	I	271 ± 3 fg	27 ± 1 c–e	97 ± 1 e–h	I
800-20	261 ± 2 de	26 ± 2 ef	101 ± 1 a	I	282 ± 8 g	30 ± 1 c–e	110 ± 4 i	I
800-30	248 ± 6 bc	25 ± 1 c–e	95 ± 3 a	I	262 ± 2 ef	34 ± 1 fg	101 ± 1 h	I
800-40	240 ± 2 ab	28 ± 2 f	98 ± 6 a	I	243 ± 3 cd	25 ± 1 b–d	90 ± 1 cd	I
800-60	247 ± 5 a–c	23 ± 1 b	92 ± 2 a	I	185 ± 1 b	25 ± 1 b–d	76 ± 1 a	II

Mean values of two extracts conducted in duplicate (2 × 2 n), SD = standard deviation. Cat. = Category. A dash in category means it could not be considered as saffron according to ISO 3632 (2011). One-way analysis of variance (ANOVA) for each column is included. Different letters within each column represent statistically significant variances, according to the Duncan test (*p* < 0.05).
